# Integrative description of *Diosaccus koreanus* sp. nov. (Hexanauplia, Harpacticoida, Miraciidae) and integrative information on further Korean species

**DOI:** 10.3897/zookeys.927.49042

**Published:** 2020-04-16

**Authors:** Byung-Jin Lim, Hyun Woo Bang, Heejin Moon, Jinwook Back

**Affiliations:** 1 Department of Taxonomy and Systematics, National Marine Biodiversity Institute of Korea, Seocheon 33662, Korea National Marine Biodiversity Institute of Korea Seocheon South Korea; 2 Mokwon University, Daejeon, 35349, Korea Mokwon University Daejeon South Korea

**Keywords:** Key, mitochondrial cytochrome c oxidase subunit I, *Parathalestris verrucosa*, *Peltidium quinquesetosum*, taxonomy, 18S ribonucleic acid

## Abstract

A new species of *Diosaccus* Boeck, 1873 (Arthropoda, Hexanauplia, Harpacticoida) was recently discovered in Korean waters. The species was previously recognized as *D. ezoensis* Itô, 1974 in Korea but, here, is described as a new species, *D. koreanus***sp. nov.**, based on the following features: 1) second inner seta on exopod of fifth thoracopod apparently longest in female, 2) outer margin of distal endopodal segment of second thoracopod ornamented with long setules in male, 3) caudal seta VII located halfway from base of rami (vs. on anterior extremity in *D. ezoensis*), and 4) sixth thoracopod with three setae in female (vs. 2 setae in *D. ezoensis*). In addition, there is also a mitochondrial COI sequence difference of more than 19.93% with *D. ezoensis* registered in NCBI. A key to *Diosaccus* species of the world is also provided, and new morphological features and DNA sequences are presented for two other harpacticoid species, *Parathalestris verrucosa* Itô, 1970 and *Peltidium quinquesetosum* Song & Yun, 1999. In order to clearly identify harpacticoids at the species level, both morphological and DNA sequence characteristics should be considered.

## Introduction

Harpacticoids (Arthropoda, Hexanauplia, Harpacticoida) are a group of benthic metazoans that are diverse in terms of both species and ecology. To date, ca 150 species of marine harpacticoids have been reported in Korean waters (Song et al. 2012). However, the diversity of harpacticoids in Korean waters is likely underestimated because many of these species have been identified on the basis of morphological characters, which are often insufficient for species identification owing to minor differences among closely-related taxa (Beheregaray and Caccone 2007; Vakati et al. 2019). In the case of *Tigriopus japonicus* Mori, 1938 collected from the Northwest Pacific Ocean, it is very difficult to identify its three cryptic species based on morphological characters, because there is no single morphological character that can distinguish among them (Karanovic et al. 2018). Several authors report species showing small morphological differences compared to the original descriptions, but have concluded that these are not sufficient for species differentiation (Chang 2007; Back and Lee 2011; Kim et al. 2011; Park and Lee 2011; Park et al. 2012; Kim et al. 2015). There is currently no clear way to distinguish between inter-species and intra-species differences.

In contrast to morphology-based taxonomy, recent advances in the cost and ease DNA sequencing and in the availability of public DNA sequence databases has facilitated the identification of numerous cryptic animal species (Hebert et al. 2003; Bhadury et al. 2006; DeSalle and Goldstein 2019), with the mitochondrial cytochrome c oxidase subunit I gene (*COI*) commonly used for species identification and the 18S ribonucleic acid gene (*18SrRNA*) commonly used for higher-level taxonomic grouping. Yet, to define new species on the basis of DNA sequences, accurate sequences of known species are needed, and few attempts have been made to assign DNA sequences to morphologically-defined harpacticoid species. Therefore, the aim of the present study is the integrative description of a newly discovered species, and to assign DNA sequences to a morphologically-defined species, and to identify previously unrecognized taxonomically informative morphological characteristics.

## Material and methods

### Sample collection

The samples were all collected from Korean waters which is part of the north-western Pacific Ocean (Table 1) and fixed in >95% ethanol. Harpacticoids were sorted from the samples using an M80 stereomicroscope (Leica, Wetzlar, Germany) and then frozen at -20 °C.

**Table 1.** Collection information of morphologically-defined harpacticoid species.

Species	Date	Locality	Gear (depth)	Specimen nos.
*Diosaccus koreanus* sp. nov.	25–07–2017	37°31'36.56"N, 130°49'41.77"E	hand net (0.5 m)	CR00247255
				CR00247256
	27–04–2018	35°18'39.0"N, 129°16'10.6"E	Grab (5 m)	CR00247257
				CR00247258
				CR00247259
				CR00247260
*Parathalestris verrucosa*	19–07–2017	36°42'36.63"N, 129°28'31.69"E	light trap (2 m)	All specimens
*Peltidium quinquesetosum*	19–07–2017	36°42'36.63"N, 129°28'31.69"E	light trap (2 m)	All specimens

### DNA extraction, amplification, sequencing, and analysis

Each specimen was rinsed in distilled water for 15 min to remove ethanol and then transferred, using a sterilized pipette tip or dissection needle, to a 1.5-mL tube that contained 20 mL Proteinase K and 180 mL ATL buffer for non-destructive DNA extraction (DNeasy Blood and Tissue Kit, Qiagen, Hilden, Germany). After the specimens were incubated for 3 h in a thermoshaker (350 rpm, 56 °C), the 200 mL of lysis buffer (Proteinase K + ATL buffer) was moved to new 1.5-mL tubes under a stereomicroscope. Each 1.5-mL specimen tube was then filled with 70% ethanol to preserve the specimens for subsequent morphological identification and description, and DNA was isolated from the buffer samples following the protocol of the DNeasy Blood and Tissue Kit.

Both *COI* and *18Sr RNA* sequences were amplified from the sample DNAs using an AccuPower HotStart PCR PreMix (Bioneer, Daejeon, South Korea), gene-specific primers (Table 2), and the amplification procedure described by Vakati et al. (2019). The resulting PCR products were sequenced in both directions using an ABI PRISM 3730XL Analyzer (Macrogen, Inc., Seoul, Korea). Sequences were assembled using Geneious 10.1.3 (Biomatters Auckland, New Zealand) (Kearse et al. 2012). Pairwise distances were calculated using the Tamura and Nei distance model (Tamura and Nei 1993) in Geneious 10.1.3. The sequences from GenBank were aligned using the Muscle algorithm integrated in Geneious 10.1.3 (Edgar 2004).

**Table 2.** Primer sequences and PCR conditions used in the present study.

Gene	References	Primer name	Primer sequence	PCR condition	Product size	Species
mt COI	Folmer et al. (1994)	LCO1490 (universal)	GGTCAACAAATCATAAAGATATTGG	94 °C, 300 s; 40 cycles × (94 °C, 60 s; 46 °C, 120 s; 72 °C, 180 s; 72 °C, 600 s)	658	*D. koreanus* sp. nov
					658	*Pa. verrucosa*
		HCO2198 (universal)	TAAACTTCAGGGTGACCAAAAAATCA			
					661	*Pe. quinquesetosum*
18S rRNA	Yamaguchi (2003)	18SF1 (universal)	TACCTGGTTGATCCTGCCAG	94 °C, 300 s; 40 cycle × (94 °C, 30 s; 50 °C, 30 s; 72 °C, 60 s); 72 °C, 420 s	1,756	*D. koreanus* sp. nov
		18SR9 (universal)	GATCCTTCCGCAGGTTCACCTAC		1,761	*Pa. verrucosa*
		18SF2 (internal)	CCTGAGAAACGGCTRCCACAT	These primers were used for primer walking to sequence over 1700 bp	1,763	*Pe. quinquesetosum*
		18SF3 (internal)	GYGRTCAGATACCRCCSTAGTT			
		18SF4 (internal)	GGTCTGTGATGCCCTYAGATGT			
		18SR6 (internal)	TYTCTCRKGCTBCCTCTCC			
		18SR7 (internal)	GYYARAACTAGGGCGGTATCTG			
		18SR8 (internal)	ACATCTRAGGGCATCACAGACC			

### Morphological characterization

After processing for molecular analysis, each specimen was dissected on several slides using lactophenol as a mounting medium and then observed using a Leica DM2500 microscope that was equipped with a drawing tube. Descriptive terminology was adopted from Huys et al. (1996).

Abbreviations used in the text are: **A1**: antennule; **A2**: antenna; **ae**: aesthetasc; **exp-1(2, 3)**: proximal (middle, distal) exopod; **enp-1(2, 3)**: proximal (middle, distal) endopod; **P1–P6**: first to sixth thoracopod; **seg-1(-5)**: first (to fifth) segment; **benp**: baseoendopod; **mxp**: maxilliped.

## Taxonomy

### Order Harpacticoida Sars, 1903

#### Family Miraciidae Dana, 1846

##### Genus *Diosaccus* Boeck, 1873

###### Diosaccus koreanus sp. nov.

Taxon classificationAnimaliaHarpacticoidaMiraciidae

83AC3306-F876-5DCF-87E3-3C898853F689

http://zoobank.org/64547C65-0584-47D1-BEDF-AC6DDD748CB6

Figs 1 , 2 , 3 , 4 , 5 , 6 , 7 , 8

####### Material examined.

***Holotype*.** Republic Of Korea ∙ Ulleungdo Island; 37°31'36.56"N, 130°49'41.77"E; 25 July 2017; B. Jinwook leg.; hand net, 0.5 m ∙ 1 ♀ (MABIK CR00247255) was dissected on 14 slides (Table 1) ∙ GenBank accession number for *COI* sequence: MN996281. ***Paratypes*.** Republic Of Korea (Table 1) ∙ 1 ♂ (MABIK CR00247257) was dissected on 8 slides and observed ∙ 4 ♀♀ (MABIK CR00247256, CR00247258 – CR00247260) were preserved in 99% alcohol ∙ GenBank accession numbers: MN996277 to MN996280 (*COI*) and MT002900 to MT002902 (*18SrRNA*).

####### Description.

**Figure 1.**
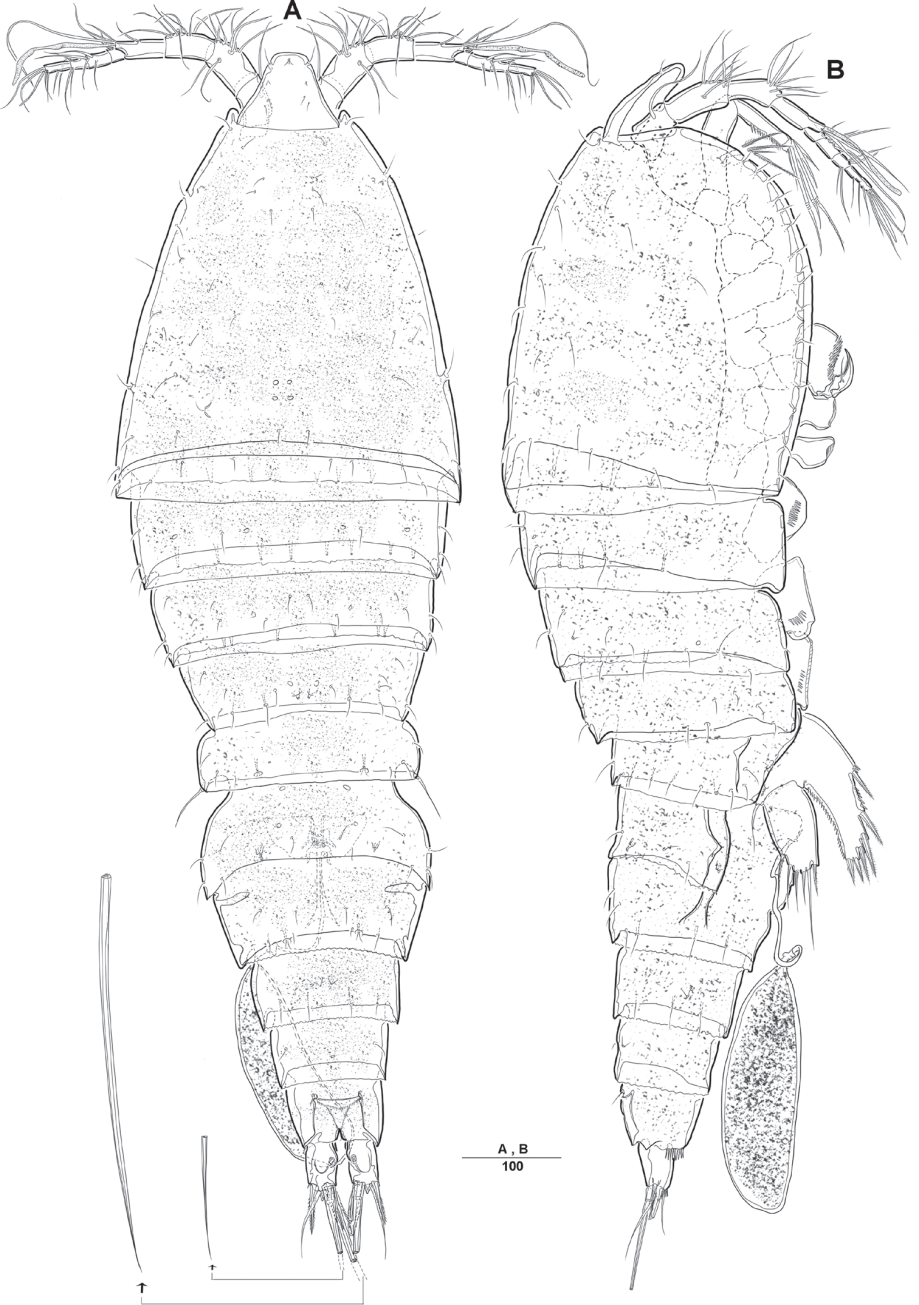
*Diosaccus koreanus* sp. nov., female **A** habitus, dorsal **B** habitus, lateral. Scale bars indicate length in µm.

**Figure 2.**
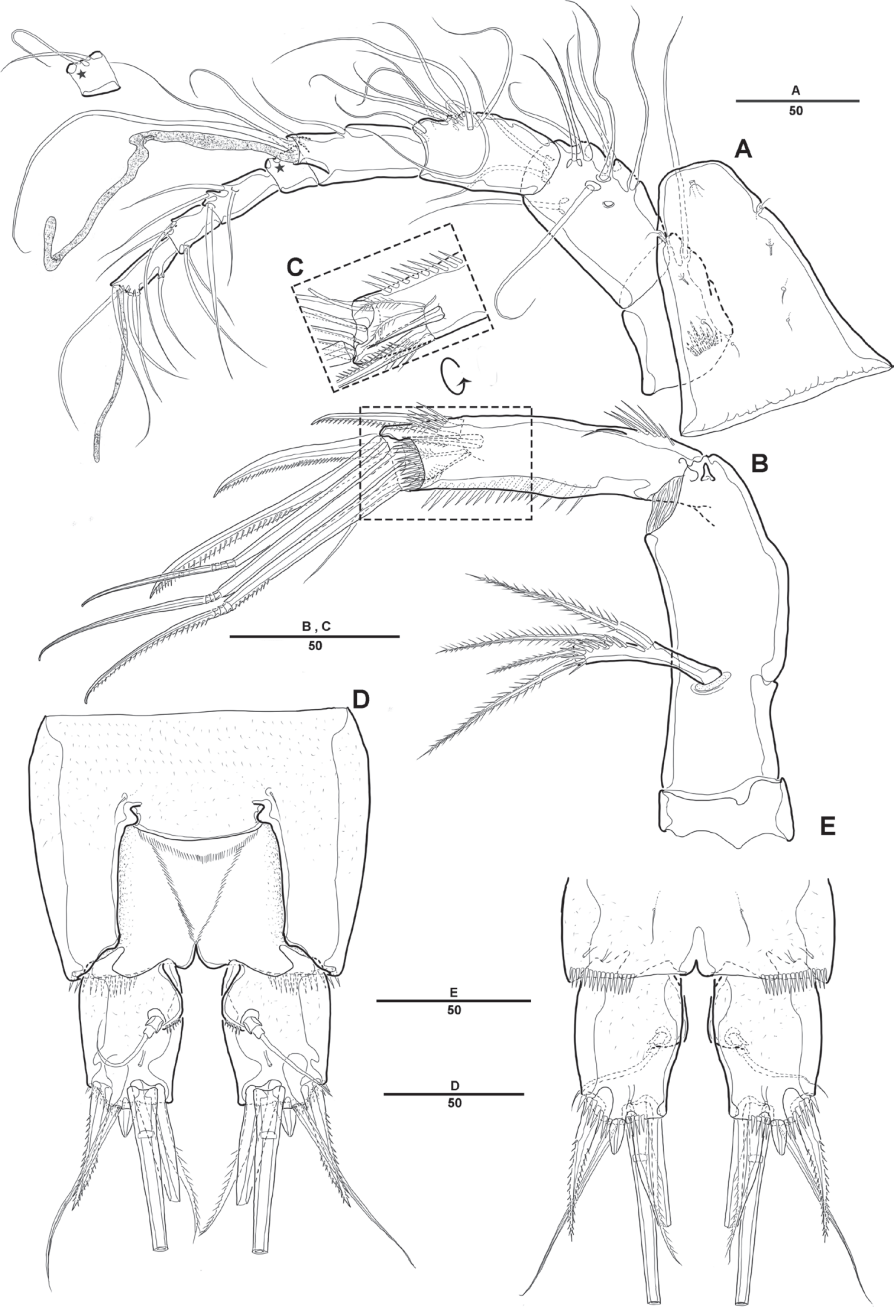
*Diosaccus koreanus* sp. nov., female **A** rostrum and antennule, dorsal **B** antenna **C** end of antennary endopod **D** caudal rami, dorsal **E** caudal rami, ventral. Scale bars indicate length in µm.

**Female. *Body*** (Figs 1, 2): Total length, from anterior margin of rostrum to posterior margin of caudal rami, 1135 μm (*N* = 5, mean = 1133 μm; Fig. 1); maximum width 340 μm, measured at distal cephalothorax; body cylindrical, not dorsoventrally depressed, and with minute dorsal sensilla; rostrum well developed, defined at base, trapezoid in shape, with round apex and 2 sensilla (Figs 1A, 2A); cephalothorax sub-triangle with sensilla and smooth margin; second and third urosomites fully fused ventrally, but with transverse ridge on dorsal and lateral surfaces indicating original segmentation (Figs 1A, B, 6B); anal operculum not well developed, with spinular tuft (Fig. 2D).

***Caudal rami*** (Fig. 2D, E): Parallel, ca 1.5 times longer than maximum width, dorsal surface with small bumps; each ramus with 7 setae: seta I strong, pinnate; setae II bare on distal corner; seta III blunt spine; setae IV and V strong; seta VI pinnate; seta VII bare, triarticulate at base.

***A1*** (Fig. 2A): Slender, 8-segmented; seg-2 longest, ca 1.2 times as long as seg-3; seg-4 with sub-cylindrical pedestal armed with aesthetasc fused at base to 1 long bare seta; armature formula: 1–[1], 2–[11], 3–[9], 4–[3 + (1+ae)], 5–[2], 6–[4], 7–[4], 8–[3+acrothek]; apical acrothek of short aesthetasc fused basally to 2 bare setae.

***A2*** (Fig. 2B, C): 3-segmented, with coxa, allobasis, and free 1-segmented enp; coxa small and bare; allobasis without abexopodal seta; exp 1-segmented, with 2 lateral and 2 apical pinnate setae; free enp with 2 pinnate setae and 2 long spines laterally and with 1 bare seta, 2 spines, and 3 geniculate setae along distal margin.

**Figure 3.**
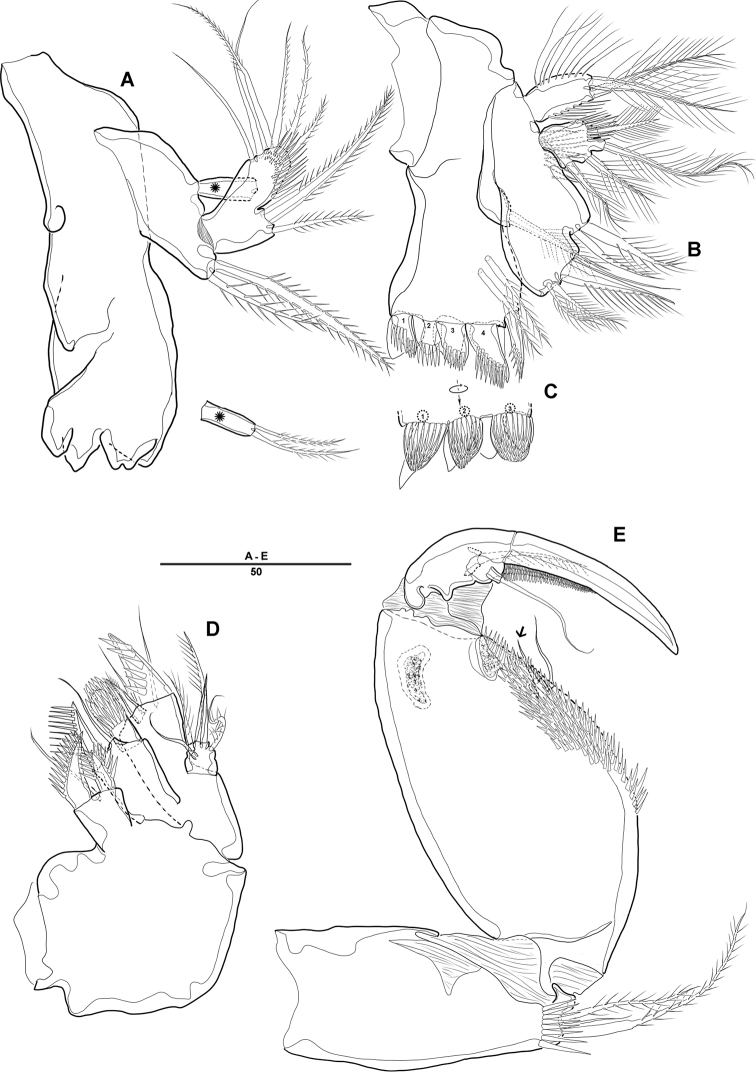
*Diosaccus koreanus* sp. nov., female **A** mandible **B** maxillule **C** shape of elements in praecoxal arthrite of maxillule **D** maxilla **E** maxilliped. Scale bars indicate length in µm.

***Mandible*** (Fig. 3A): Gnathobase with several blunt teeth; palp basis with 2 inner pinnate setae; exp 1-segmented with 2 pinnate distal setae; enp with 2 lateral and 6 distal setae.

***Maxillule*** (Fig. 3B, C): Praecoxa trapezoidal in shape, without ornamentation; arthrite well developed, with 2 juxtaposed setae near midpoint of anterior surface, 4 strong teeth-like spines and 3 tuft spines along distal margin; coxa fused with cylindrical endite, with 1 pinnate seta; basis fused with endite, with 1 bare and 5 pinnate setae; exp 1-segmented, with 2 pinnate setae distally; enp 1-segmented, with 4 pinnate setae along distal margin.

***Maxilla*** (Fig. 3D): Syncoxa with 2 endites; proximal endite with 2 strong spines and 1 bare seta among distal margin; second endite with 1 strong spine, 1 bare seta, and 1 tuft-like seta; allobasis developed into cylindrical process, with 2 strong spines and 2 bare setae; enp 1-segmented, with 2 bare and 3 pinnate setae.

***Mxp*** (Fig. 3E): 4-segmented, with syncoxa, basis, and 2-segmented enp; syncoxa with 2 pinnate setae distally; basis elongate and robust, with 2 small bare setae (Fig. 3E, arrow) and roughly ornamented with rows of spinules along inner margin; enp-1 with 1 bare and 1 pinnate setae; enp-2 forming strong claw ornamented with row of spinules among inner proximal half.

**Figure 4.**
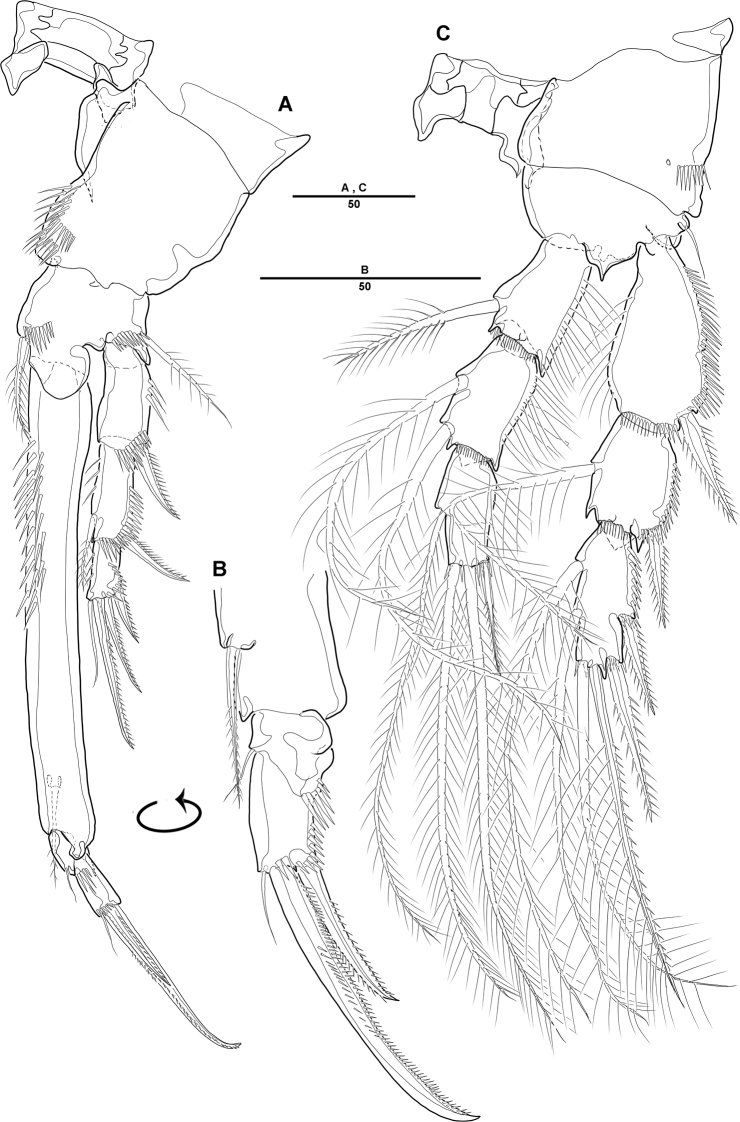
*Diosaccus koreanus* sp. nov., female **A** first thoracopod **B** middle and distal endopods of first thoracopod **C** second thoracopod. Scale bars indicate length in µm.

**Figure 5.**
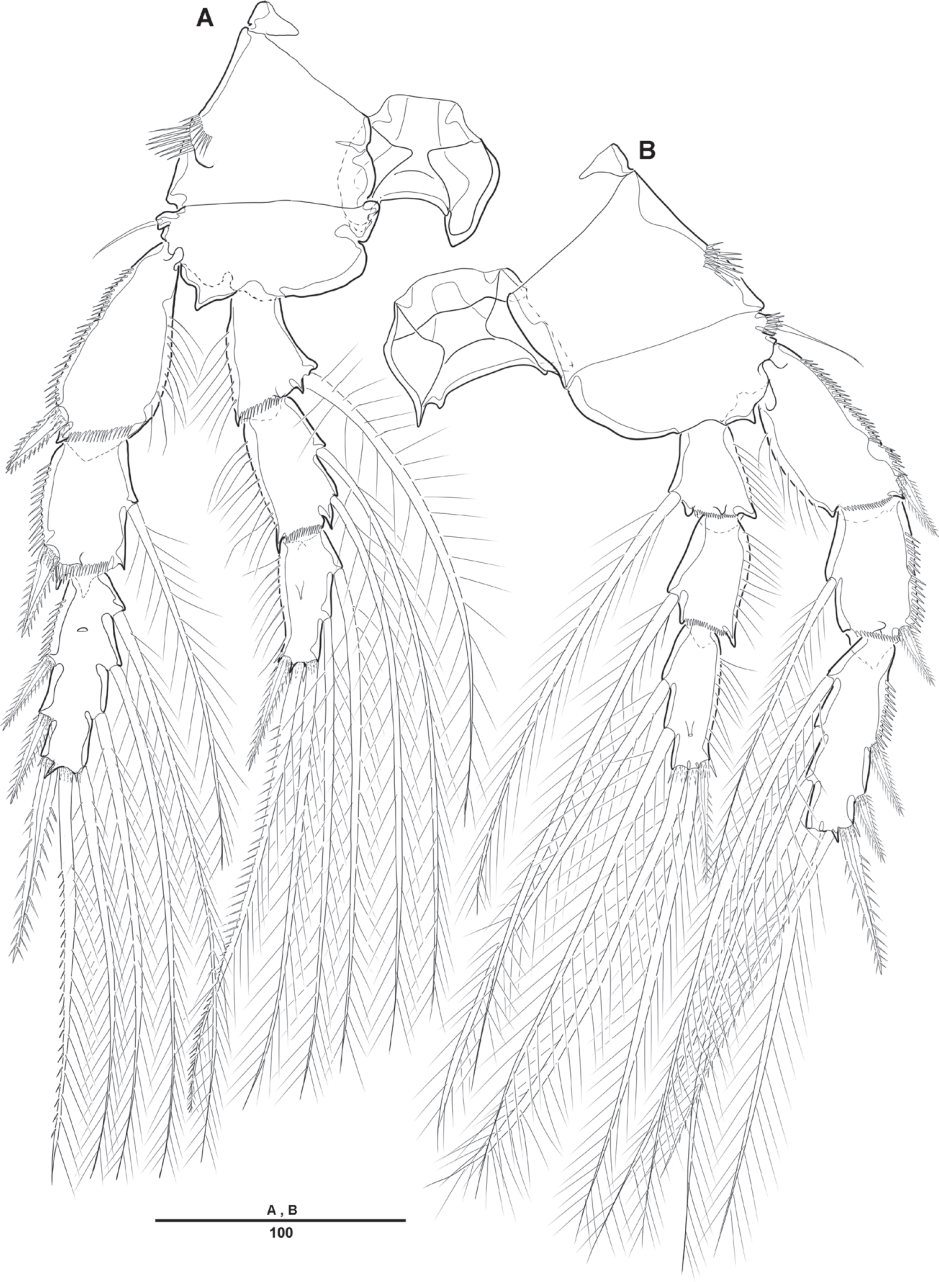
*Diosaccus koreanus* sp. nov., female **A** third thoracopod **B** fourth thoracopod. Scale bars indicate length in µm.

***Swimming legs*** (Figs 4, 5): Biramous; P1–P4 with coxa, basis, and 3-segmented exp and enp; each ramus ornamented with setules or spinules along outer margins as figured.3

***P1*** (Fig. 4A, B): Coxa ornamented with inner spinules; basis with 1 outer and 1 inner pinnate setae; exp-1 with 1 outer spine; exp-2 with 1 outer spine and 1 inner pinnate seta; exp-3 with 3 spines and 1 bare seta; enp-1 ornamented with row of spinules on inner proximal half, ca 2 times longer than exp, with 1 pinnate seta; enp-2 with 1 small bare seta on inner distal corner, enp-3 with 2 strong spines distally and 1 bare seta near inner distal corner.

***P2*** (Fig. 4C): Coxa ornamented with row of spinules on outer margin; basis with 1 outer bare seta near distal corner; exp-1 with 1 outer spine, ornamented with a row of long setules along inner margin; exp-2 with 1 outer spine and 1 inner pinnate seta, ornamented with row of setules along outer margin; exp-3 with 2 outer spines and 2 apical and 2 inner pinnate setae; enp-1 with 1 inner pinnate seta, ornamented with long setules along outer margin; enp-2 with 2 pinnate inner setae; enp-3 with 1 outer, 2 distal, and 1 inner pinnate setae.

***P3–P4*** (Fig. 5A, B): Coxa ornamented with rows of spinules on outer margin; basis with 1 outer bare seta near distal corner; exp-1 with 1 outer spine, ornamented with row of long setules along inner margin; exp-2 with 1 outer spine and 1 inner pinnate seta, ornamented with row of spinules along outer margin; exp-3 with 3 outer spines, 2 apical and 3 inner pinnate setae; enp-1 with 1 inner seta, ornamented with long setules among outer margin; enp-2 with 2 inner pinnate setae [P3] or 1 inner pinnate seta [P4]; enp-3 with 1 outer spine, 2 apical pinnate and 2 inner pinnate setae.

Armature formulae as follows:

**Table:** 

	Exopod	Endopod
P1	0.1.112	1.1.120
P2	0.1.222	1.2.121
P3	0.1.323	1.2.221
P4	0.1.323	1.1.221

**Figure 6.**
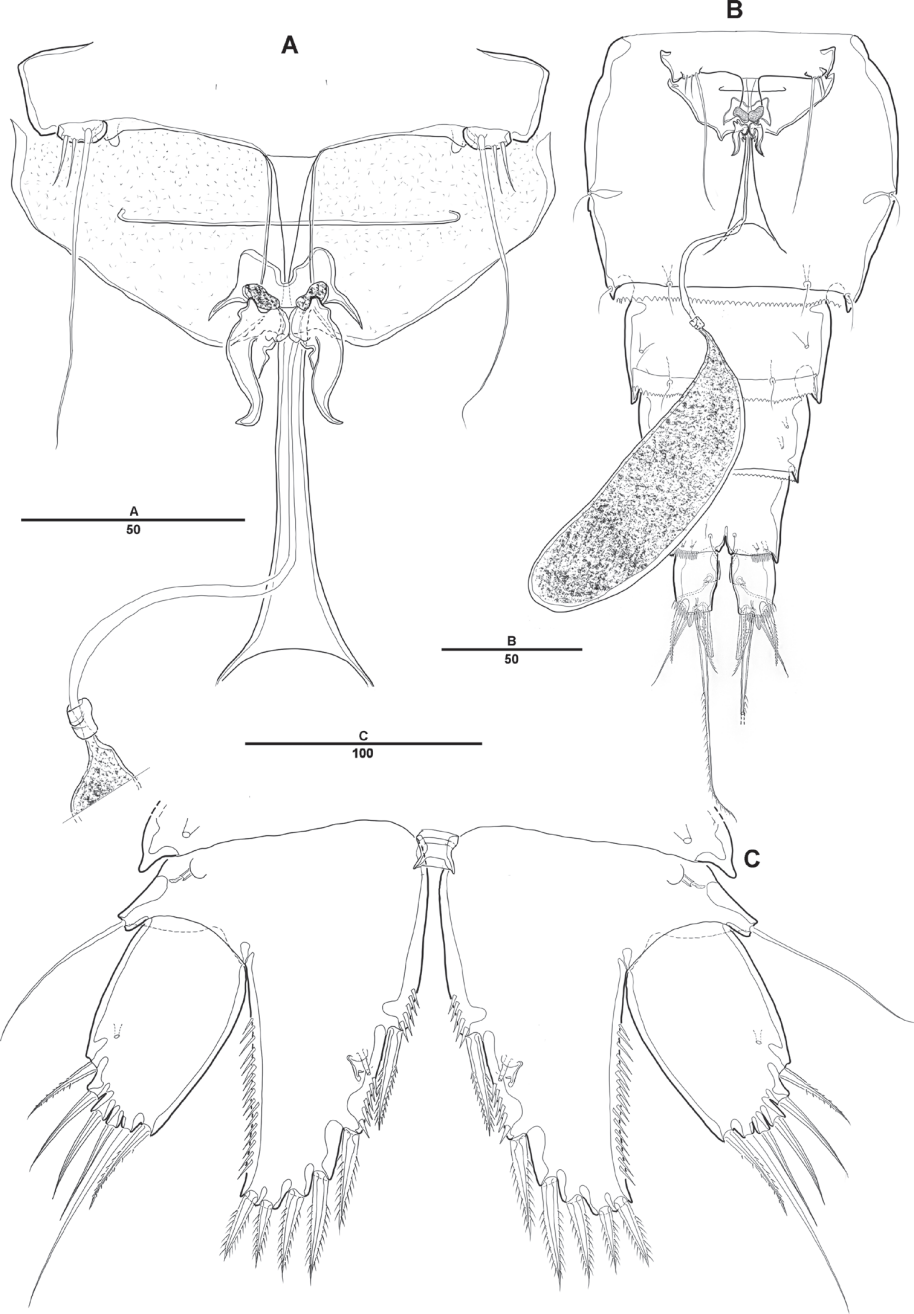
*Diosaccus koreanus* sp. nov., female **A** sixth thoracopod and genital field **B** urosomites, ventral **C** fifth thoracopod. Scale bars indicate length in µm.

***P5*** (Fig. 6C): Defined at supporting somite; each side of endopodal lobe separated, with 6 spine-like setae; exp with 6 setae, second inner element longest.

***P6*** (Fig. 6A, B): Fused with supporting somite, with 3 bare setae, innermost seta longest.

**Figure 7.**
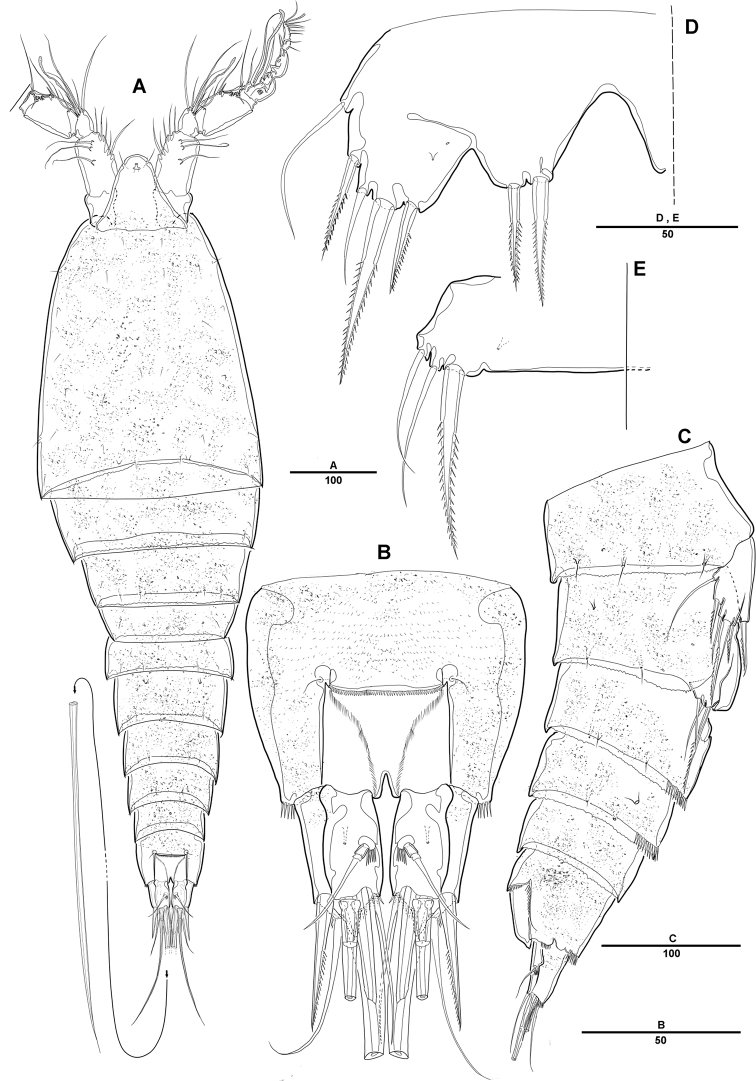
*Diosaccus koreanus* sp. nov., male **A** habitus, dorsal **B** caudal rami, dorsal **C** urosomites, lateral **D** fifth thoracopod **E** sixth thoracopod. Scale bars indicate length in µm.

**Male. *Body*** (Fig. 7A): Total length, from anterior margin of rostrum to posterior margin of caudal rami, 880 μm; maximum width 262 μm, measured at distal cephalothorax; general body shape, ornamentation, and sensilla pattern almost identical to those of female, but with sexual dimorphisms observed in A1, P1, P2, P5, P6, and genital somites.

**Figure 8.**
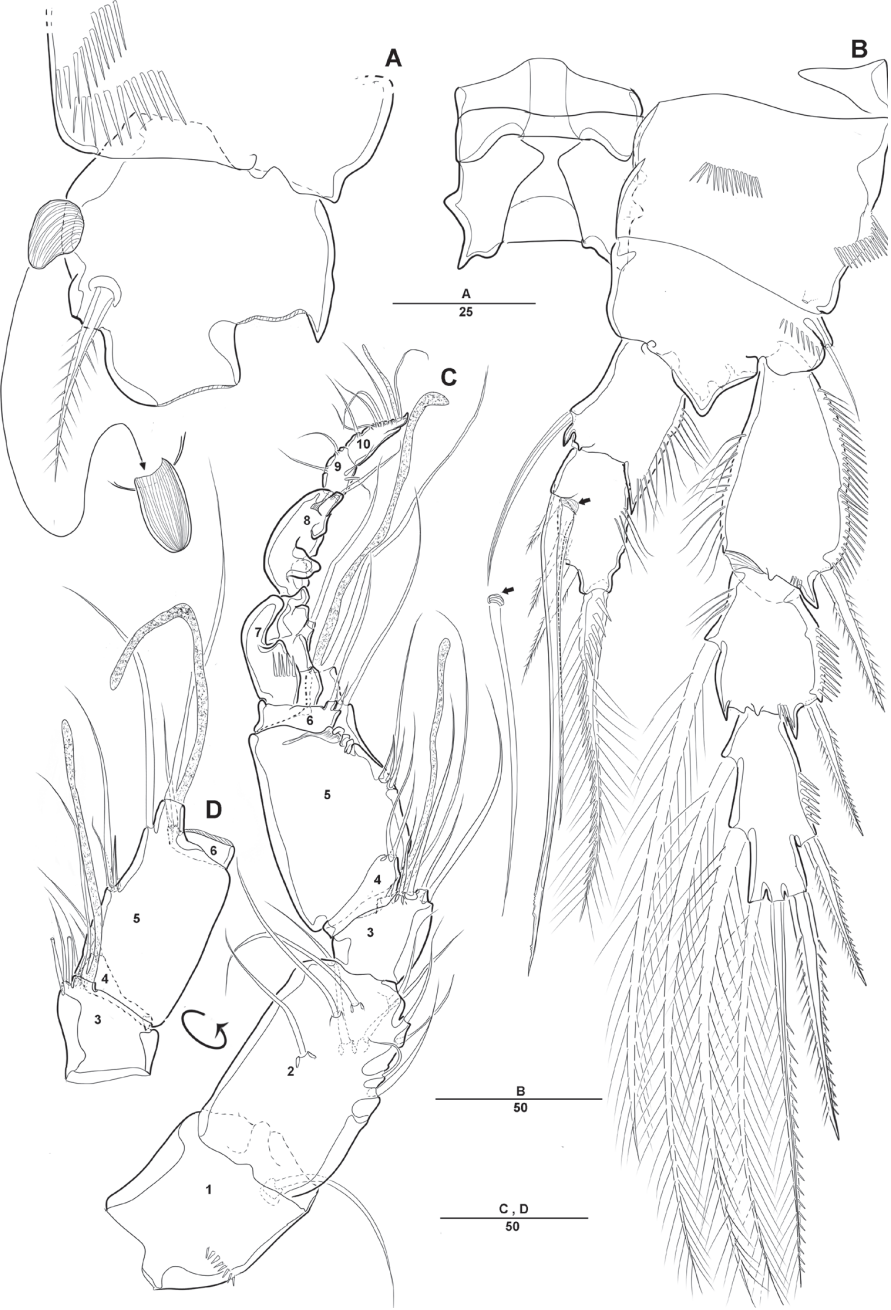
*Diosaccus koreanus* sp. nov., male **A** base of first thoracopod **B** second thoracopod **C** antennule **D** antennule segments 3–6. Scale bars indicate length in µm.

***A1*** (Fig. 8C, D): Subchirocer 10-segmented, robust; seg-3 with aesthetasc fused at base to 1 bare seta; seg-5 swollen, with aesthetasc fused at base to 1 bare seta; armature formula: 1–[1], 2–[10], 3– [4+(1+ae)], 4–[2], 5–[4+(1+ae)], 6–[2 bare], 7–[1], 8–[1], 9–[4], 10–[5+(1+ae)].

***P1*** (Fig. 8A): General shape of P1 similar to that of female, except basis; basis with 1 outer pinnate seta and 1 wrinkled process near base of outer seta.

***P2*** (Fig. 8B): Enp 2-segmented; enp-1 with 1 inner bare seta and ornamented with row of long setules along outer margin; enp-2 with 1 inner bare seta on small disk (Fig. 8B, arrow) of which middle inner edge and 1 longest bare seta, 3 pinnate inner setae, and 1 strong spinulose seta apically.

***P5*** (Fig. 7D): Fused medially; plate of benp fused each side; basal part with 1 bare seta; endopodal lobe with 2 spinulose pinnate setae; exp fused at base, with 3 spinulose setae and 1 bare seta.

***P6*** (Fig. 7E): Fused at base, with 2 bare and 1 spinulose setae.

####### Etymology.

Species name refers to the type locality (i.e., Republic of Korea).

####### DNA sequences.

In regards to pairwise distances (Tamura-Nei distance) among the 582-bp *COI* sequences, *D. koreanus* sp. nov. exhibited intra-specific variation of 0–2.28%, and inter-specific distances of 19.42–22.34% were observed among all three *Diosaccus* species (Table 3). In regards to the *18SrRNA* sequences, intra- and inter-specific variations of 0% and 1.46–8.55% were observed (Table 4).

**Table 3.** Pairwise distances (Tamura-Nei distance) between *COI* sequences from species in genus *Diosaccus*. Numbers in parentheses indicate the Genbank accession numbers.

		1	2	3	4	5	6	7	8
1	*D. koreanus* sp. nov. (CR00247255, CR00247258)								
2	*D. koreanus* sp. nov. (CR00247256)	1.52							
3	*D. koreanus* sp. nov. (CR00247257 CR00247260)	0.91	0.91						
4	*D. koreanus* sp. nov. (CR00247259)	2.28	1.67	1.67					
5	*D. ezoensis* (KR049013)	19.93	20.62	20.62	20.79				
6	*D. spinatus* (MH242730)	20.36	21.28	21.28	22.04	19.76			
7	*D. spinatus* (MH242731)	20.67	21.59	21.59	22.34	19.42	1.06		
8	*D. spinatus* (HQ966504)	20.06	20.97	20.97	21.73	19.93	1.06	0.61	

**Table 4.** Pairwise distances (Tamura-Nei distance) based on 1,756 bp between *18SrRNA* sequences from species in genus *Diosaccus*.

	**Species (Genbank accession number)**	**1**	**2**	**3**
1	*Diosaccus koreanus* sp. nov (MT002900 – MT002902)			
2	*D. ezoensis* (KR048740)	1.46		
3	*Diosaccus* sp. (EU380290)	7.24	8.55	

#### Family Thalestridae Sars G.O., 1905

##### Genus *Parathalestris* Brady & Robertson D., 1873

###### Parathalestris verrucosa

Taxon classificationAnimaliaHarpacticoidaThalestridae

Itô, 1970

AE712B6C-60D7-5621-8D34-000C66B9AAB7

**Figure 9.**
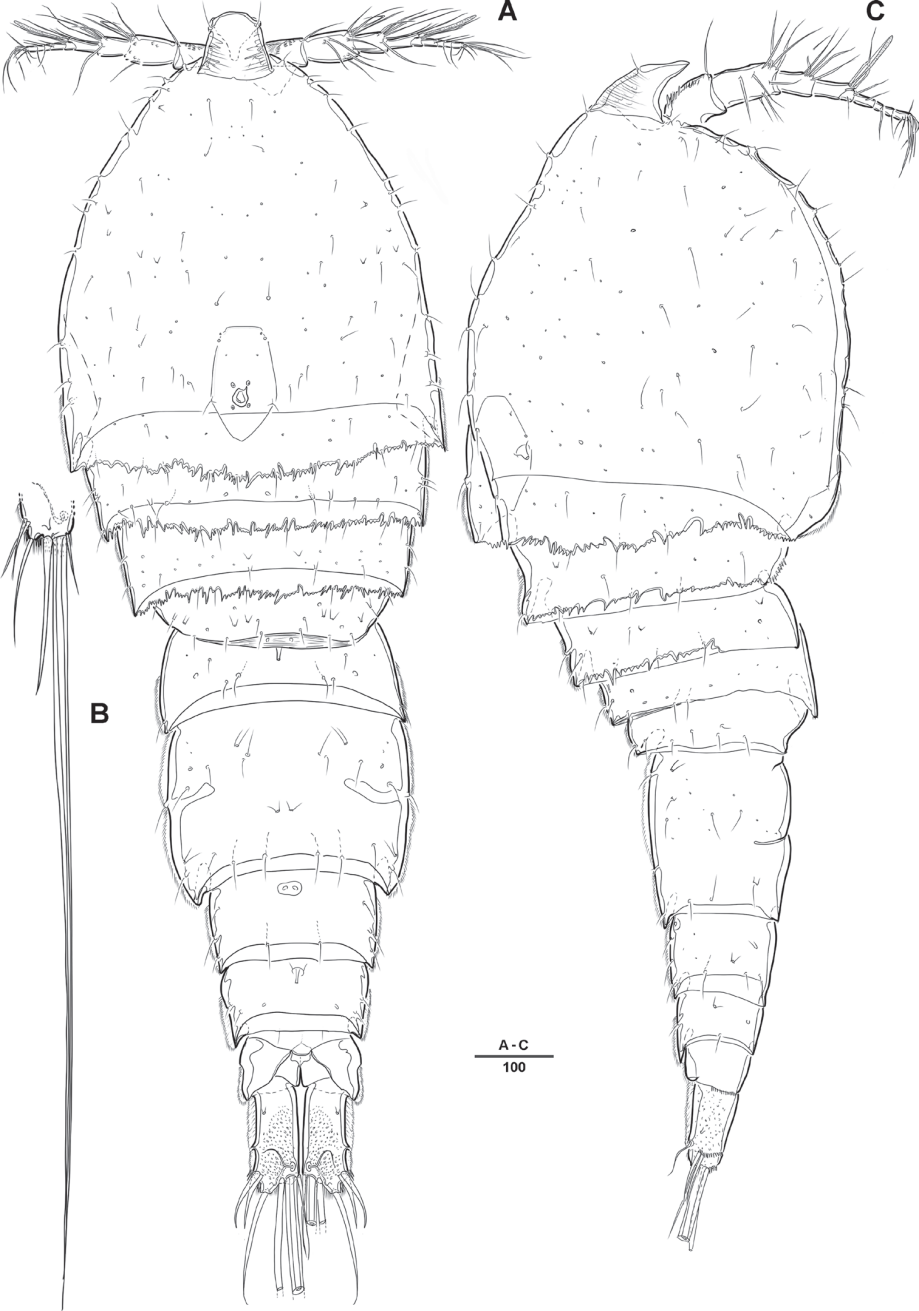
*Parathalestris verrucosa* Itô, 1970, female **A** habitus, dorsal **B** end of caudal rami **C** habitus, lateral. Scale bars indicate length in µm.

**Figure 10.**
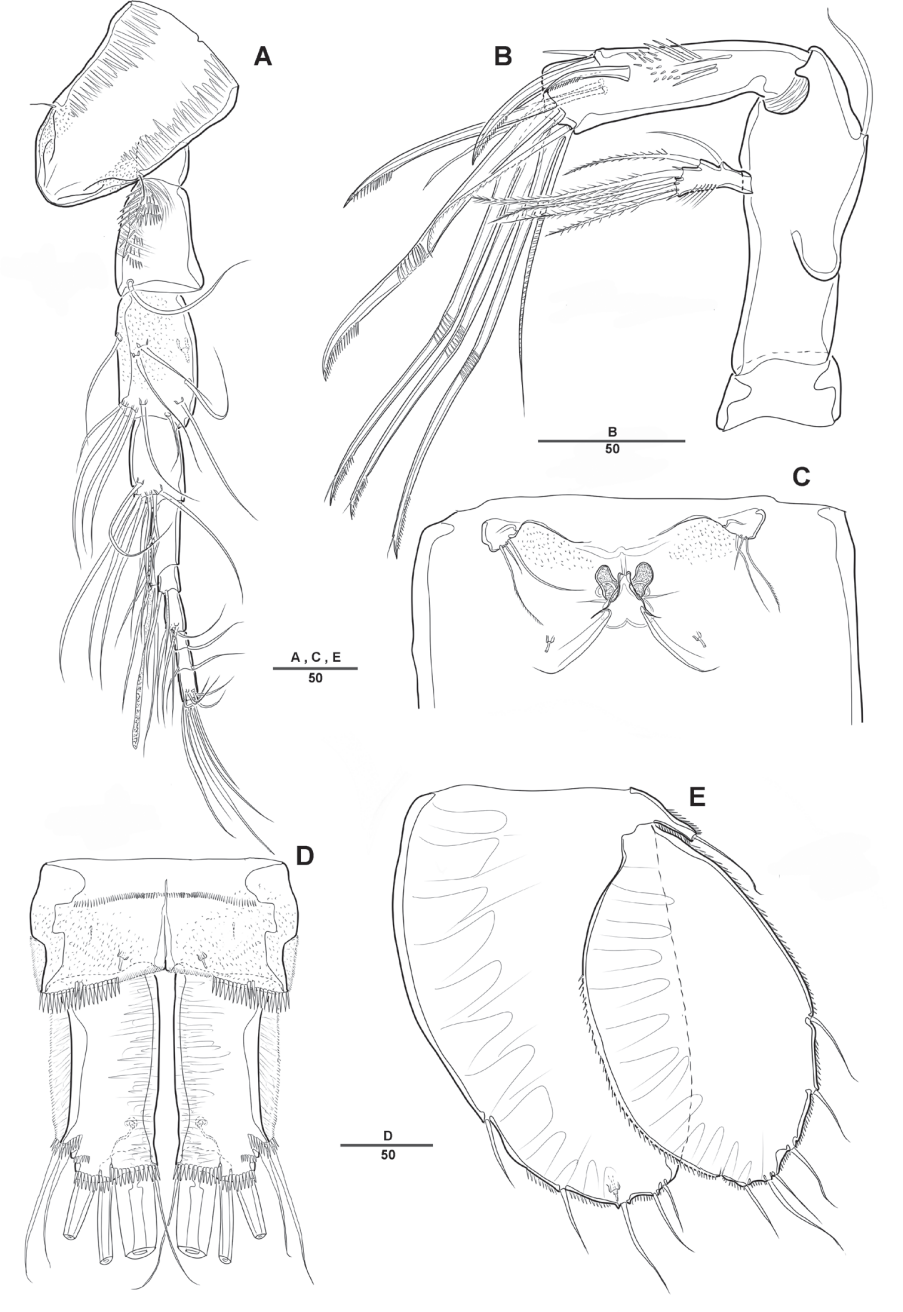
*Parathalestris verrucosa* Itô, 1970, female **A** rostrum and antennule **B** antenna **C** sixth thoracopod and genital field **D** caudal rami, ventral **E** fifth thoracopod. Scale bars indicate length in µm.

**Figure 11.**
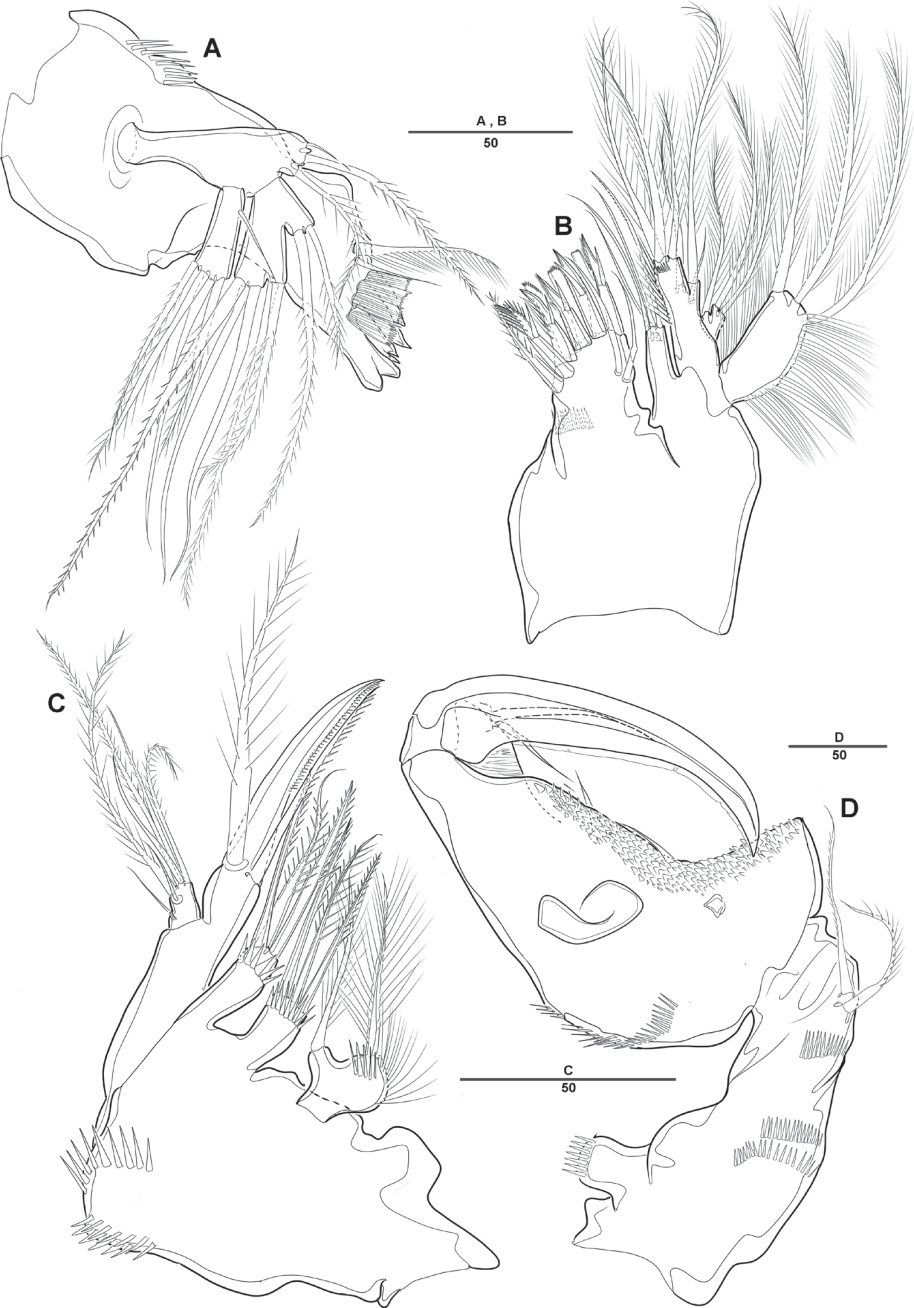
*Parathalestris verrucosa* Itô, 1970, female **A** mandible **B** maxillule **C** maxilla **D** maxilliped. Scale bars indicate length in µm.

**Figure 12.**
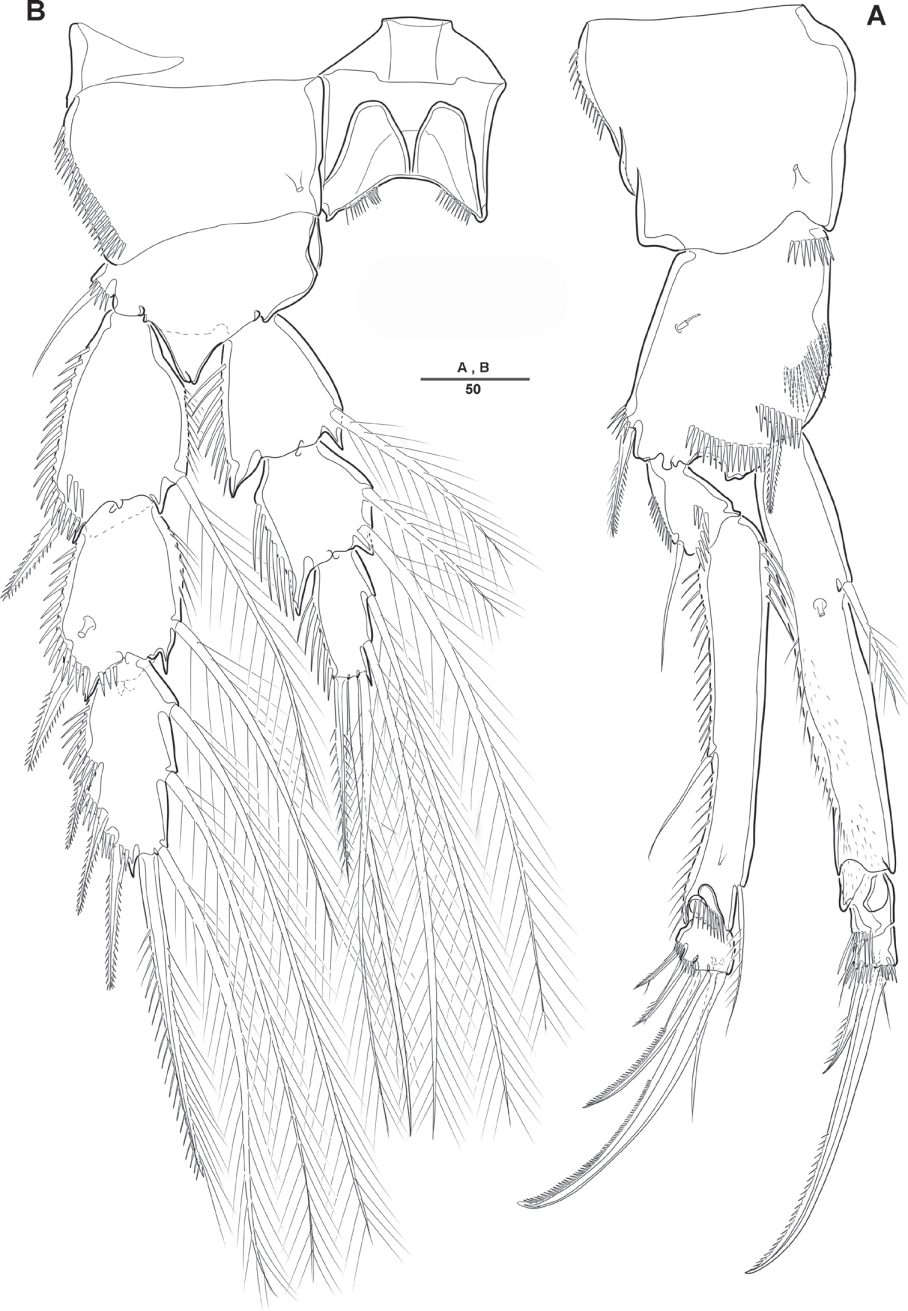
*Parathalestris verrucosa* Itô, 1970, female **A** first thoracopod **B** second thoracopod. Scale bars indicate length in µm.

**Figure 13.**
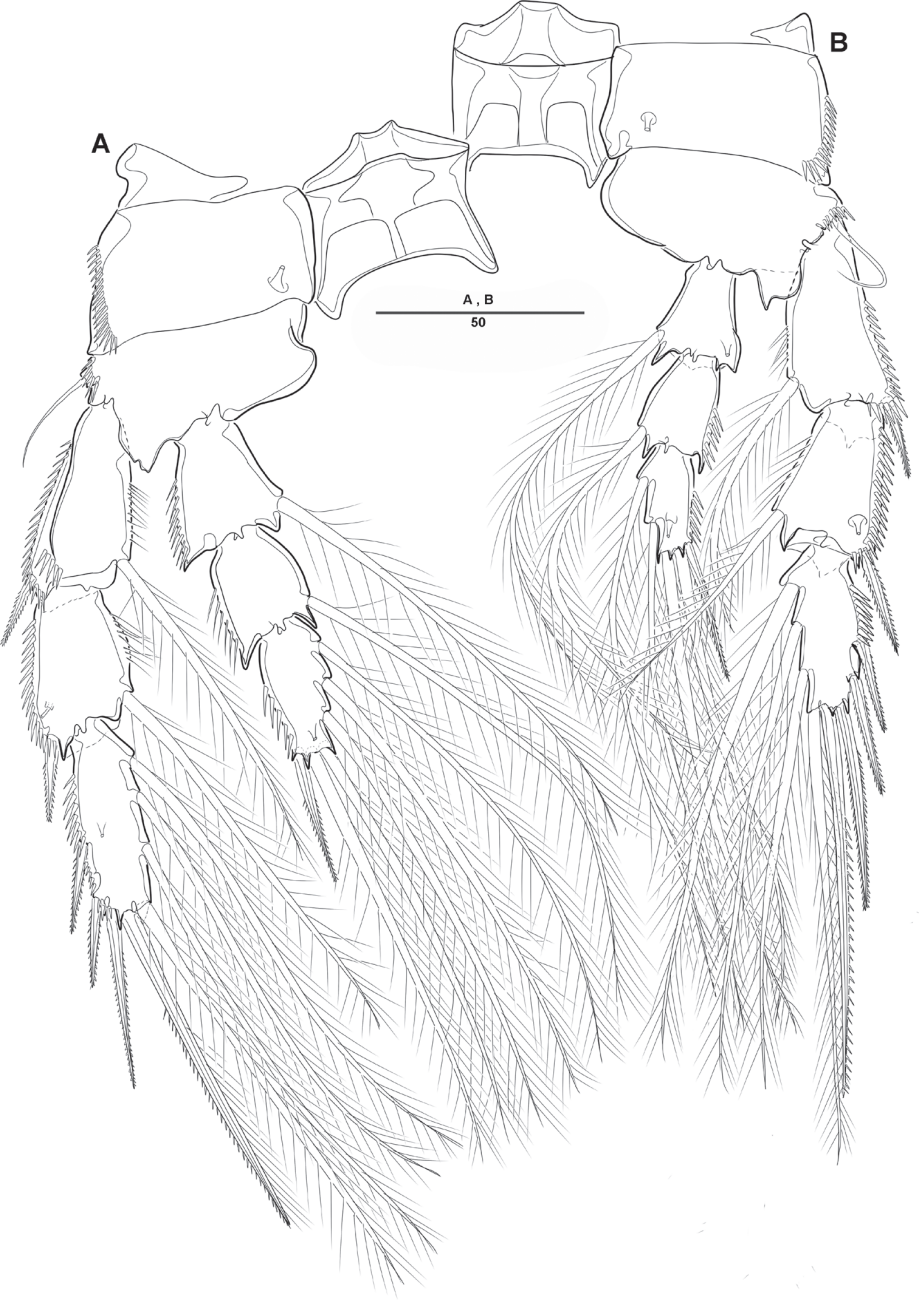
*Parathalestris verrucosa* Itô, 1970, female **A** third thoracopod **B** fourth thoracopod. Scale bars indicate length in µm.

**Figure 14.**
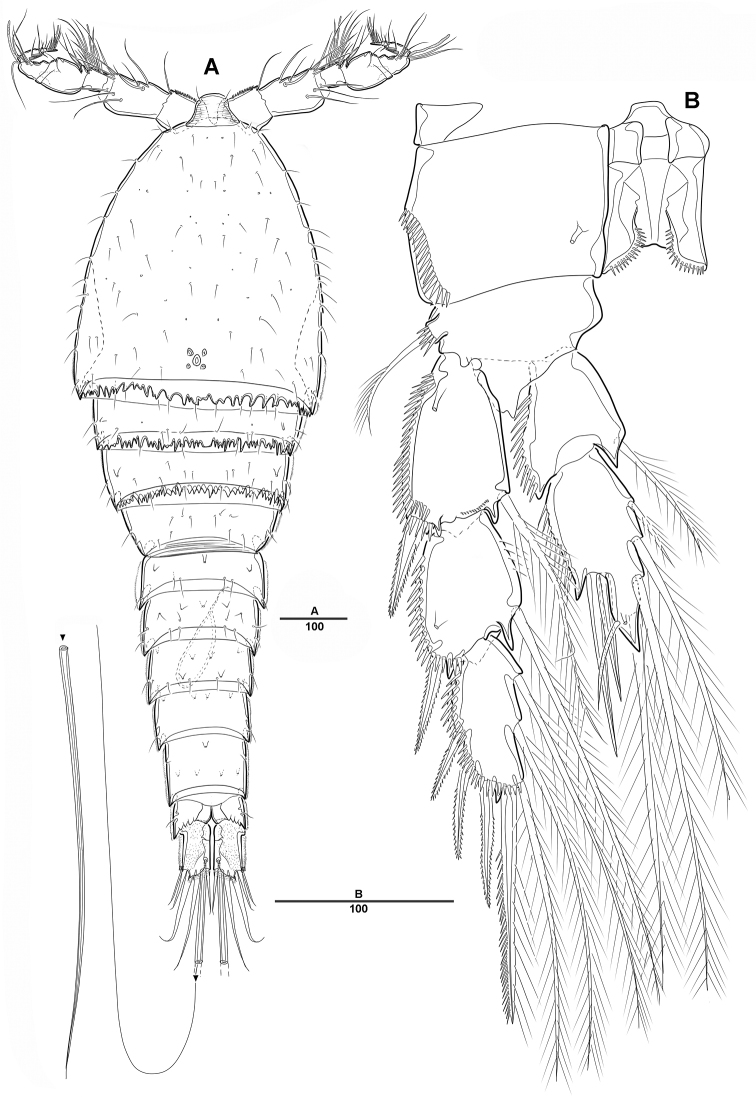
*Parathalestris verrucosa* Itô, 1970, male **A** habitus, dorsal **B** second. Scale bars indicate length in µm.

**Figure 15.**
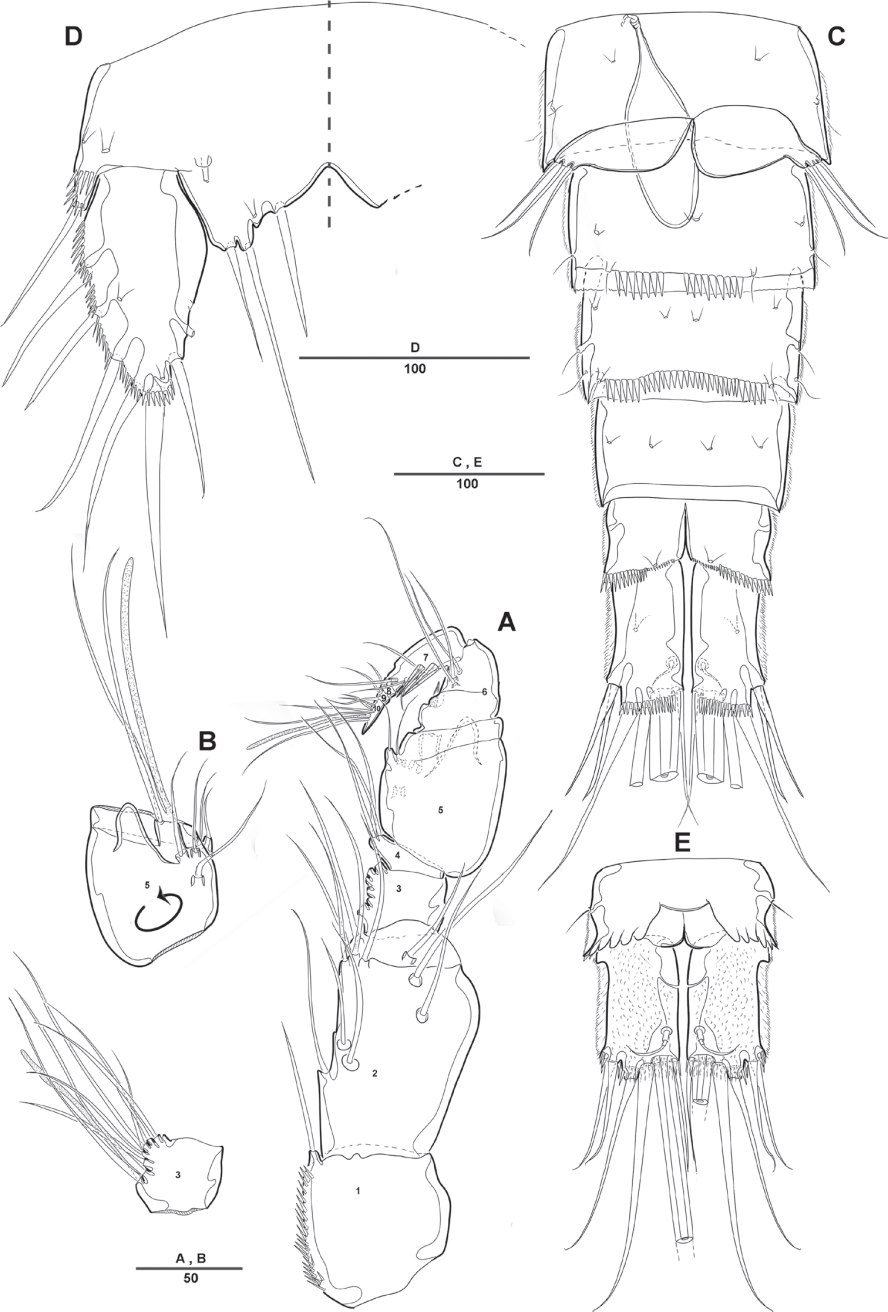
*Parathalestris verrucosa* Itô, 1970, male **A** antennule **B** antennule segments 3 and 5 **C** urosomites, ventral **D** fifth thoracapod **E** caudal rami, dorsal. Scale bars indicate length in µm.

Figs 9 , 10 , 11 , 12 , 13 , 14 , 15

####### Material examined.

Republic Of Korea ∙ 1 ♀ (MABIK CR00246555) was dissected on 13 slides ∙ 1 ♂ (MABIK CR00246552) was dissected on 9 slides ∙ 11 ♀♀ (MABIK CR00246553, CR00246554, CR00246556 to CR00246560, CR00246562 to CR00246565) and 1 ♂ (MABIK CR00246561) were preserved in 99 % alcohol ∙ GenBank accession numbers: MN996282 to MN996293 (*COI*) and MT002906 to MT002909 (*18SrRNA*).

####### Description.

*Parathalestris verrucosa* Itô, 1970 (p. 211–218, Figs 1–4), see also Chang and Song (1997).

####### Note.

Chang and Song (1997) reported that *P. verrucosa* collected from Korea differed from Itô’s description in regards to three characteristics (length of caudal rami, segmentation of A2 exp, and presence of rows of spines along posteroventral margin), and the specimens analyzed in the present study also varied in this manner. In particular, the base of the second lateral seta of the A2 exp was protruding and could be seen as two segments, depending on the angle. In addition, the male specimens analyzed in the present study also differed from Itô’s original description in regards to A1 segmentation. More specifically, the A1 of Itô’s specimen possessed a small seg-3 and swollen seg-4, whereas that of the present study’s specimens possessed small seg-3 and seg-4 and a swollen seg-5.

#### Family Peltidiidae Claus, 1860

##### Genus *Peltidium* Philippi, 1839

###### Peltidium quinquesetosum

Taxon classificationAnimaliaHarpacticoidaPeltidiidae

Song & Yun, 1999

3351DC31-6C5B-511A-8E33-1259687C2A1F

**Figure 16.**
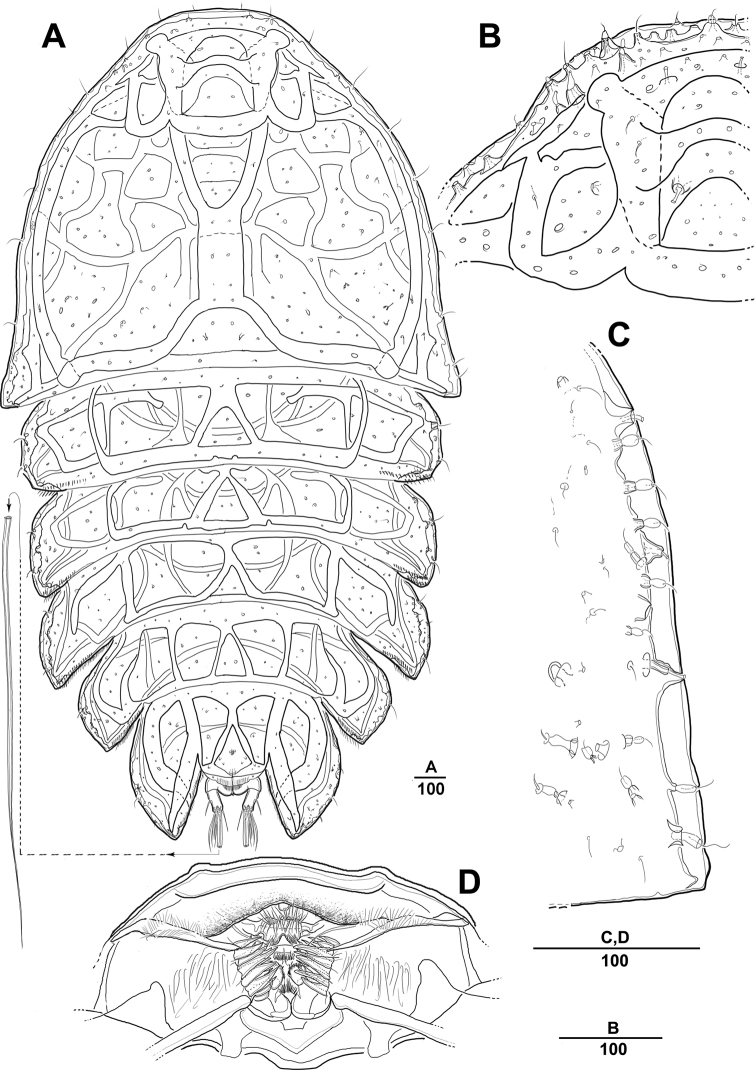
*Peltidium quinquesetosum* Song & Yun, 1999, female **A** habitus, dorsal **B** anterior tip of cephalic shield **C** lateral margin of cephalic shield **D** rabrum. Scale bars indicate length in µm.

**Figure 17.**
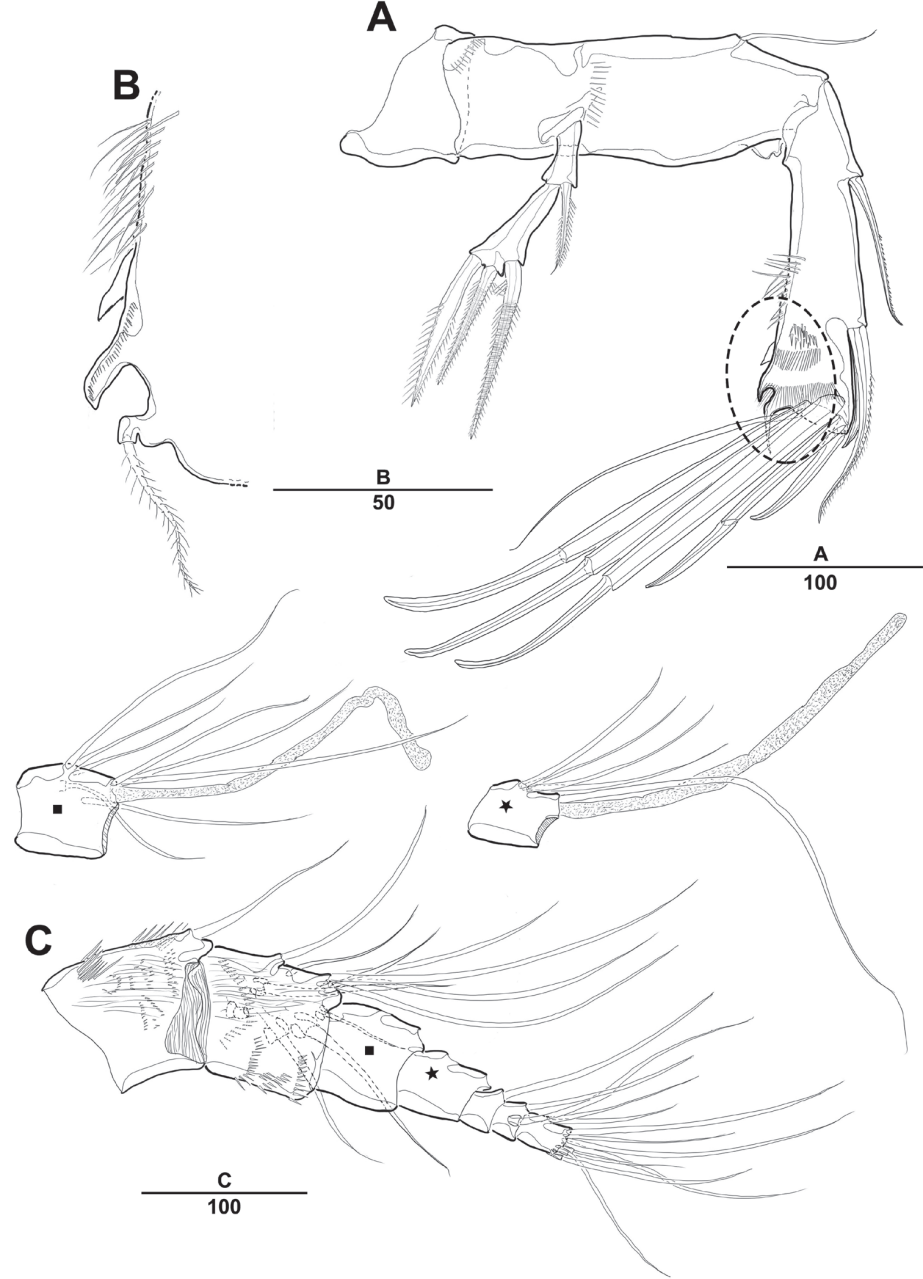
*Peltidium quinquesetosum* Song & Yun, 1999, female **A** antenna **B** end of antennary endopod **C** antennule. Scale bars indicate length in µm.

**Figure 18.**
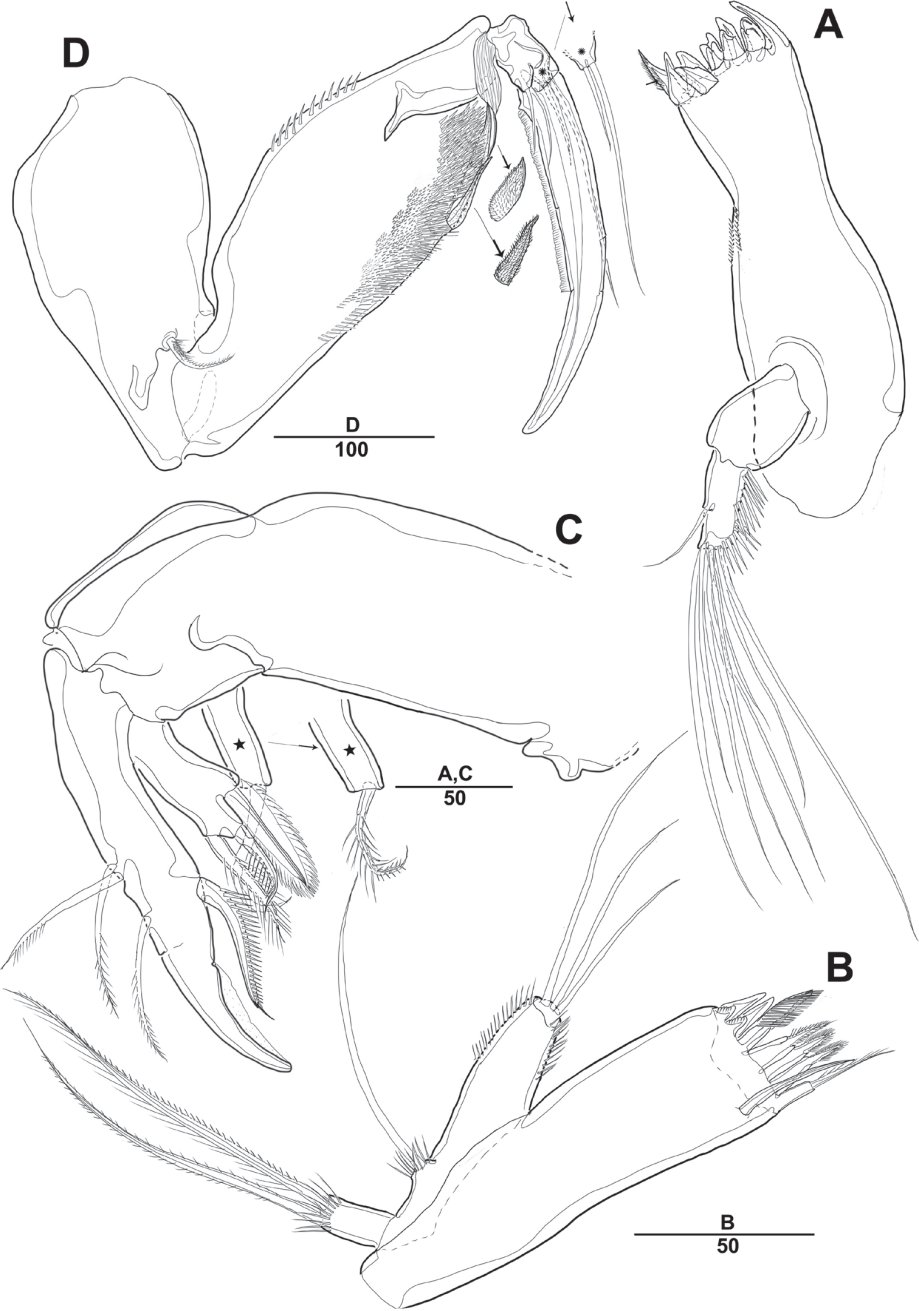
*Peltidium quinquesetosum* Song & Yun, 1999, female **A** mandible **B** maxillule **C** maxilla **D** maxilliped. Scale bars indicate length in µm.

**Figure 19.**
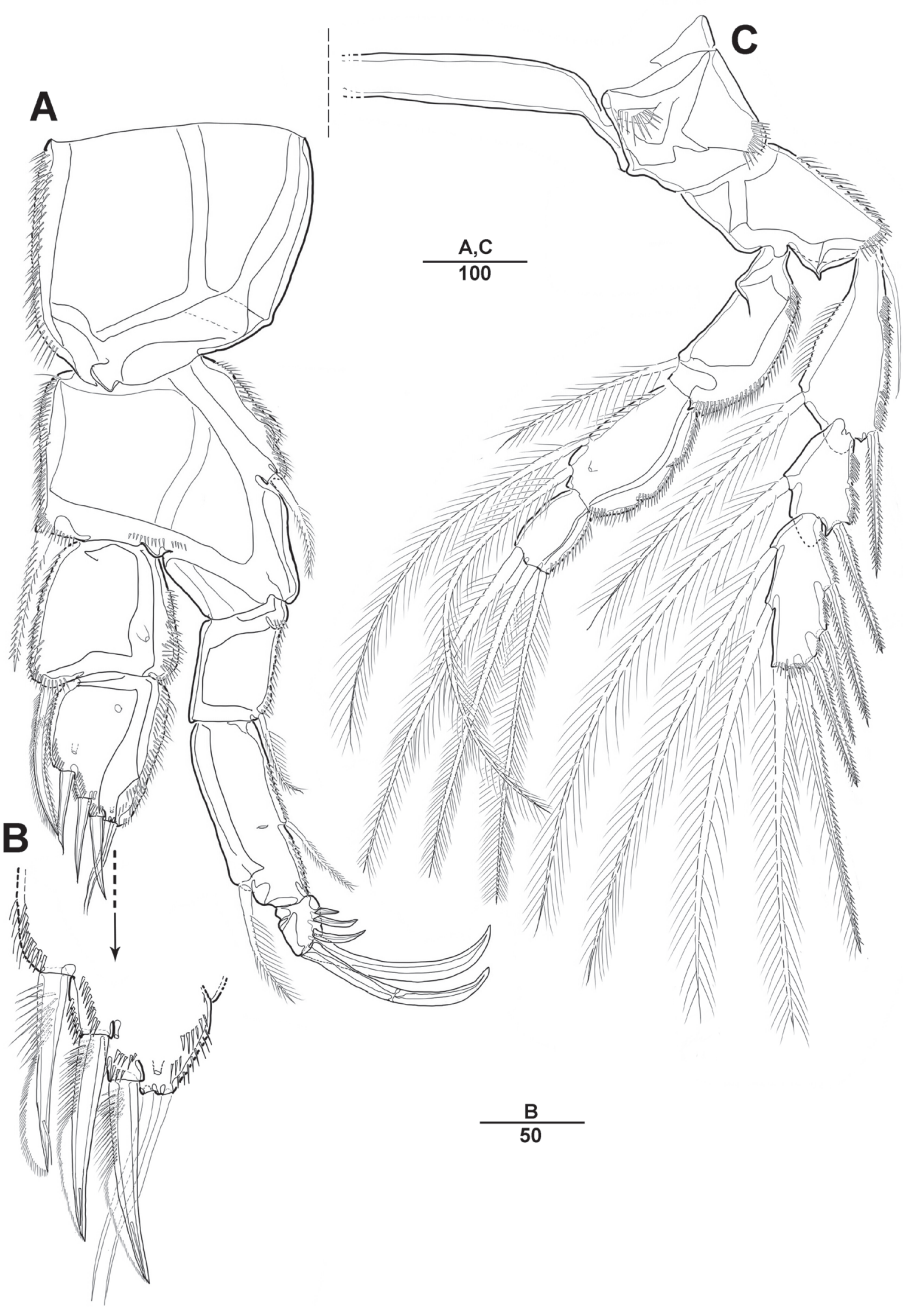
*Peltidium quinquesetosum* Song & Yun, 1999, female **A** first thoracapod **B** shape of setae on second endopod in first thoracapod **C** second thoracapod. Scale bars indicate length in µm.

**Figure 20.**
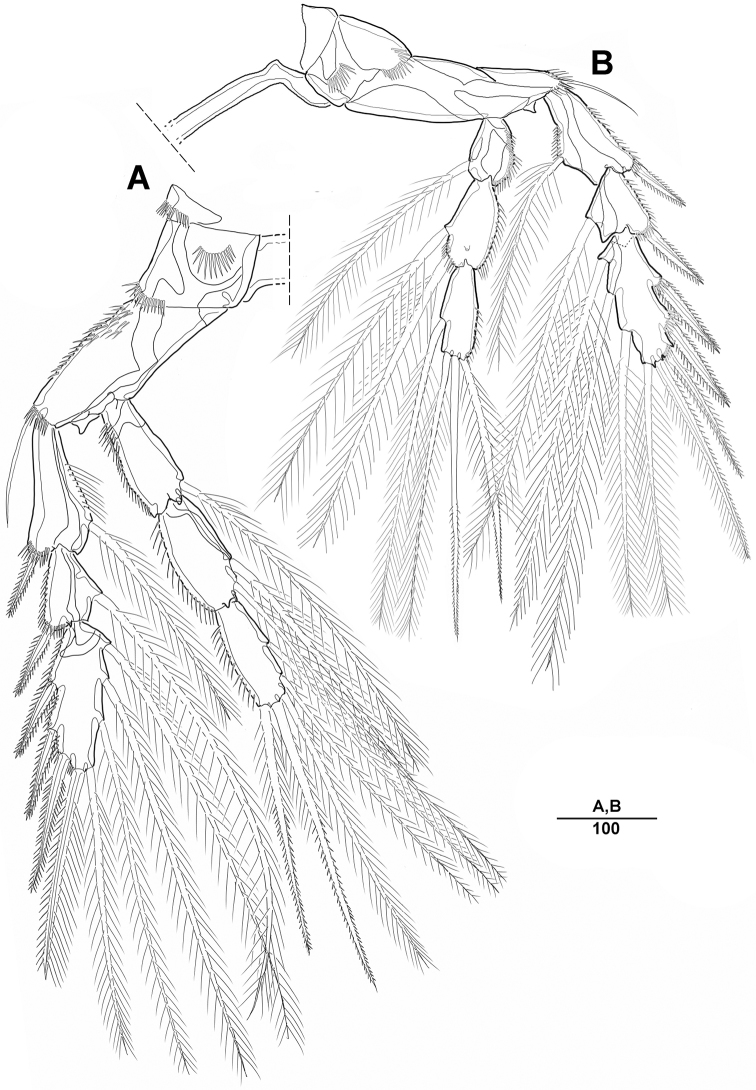
*Peltidium quinquesetosum* Song & Yun, 1999, female **A** third thoracapod **B** fourth thoracapod. Scale bars indicate length in µm.

**Figure 21.**
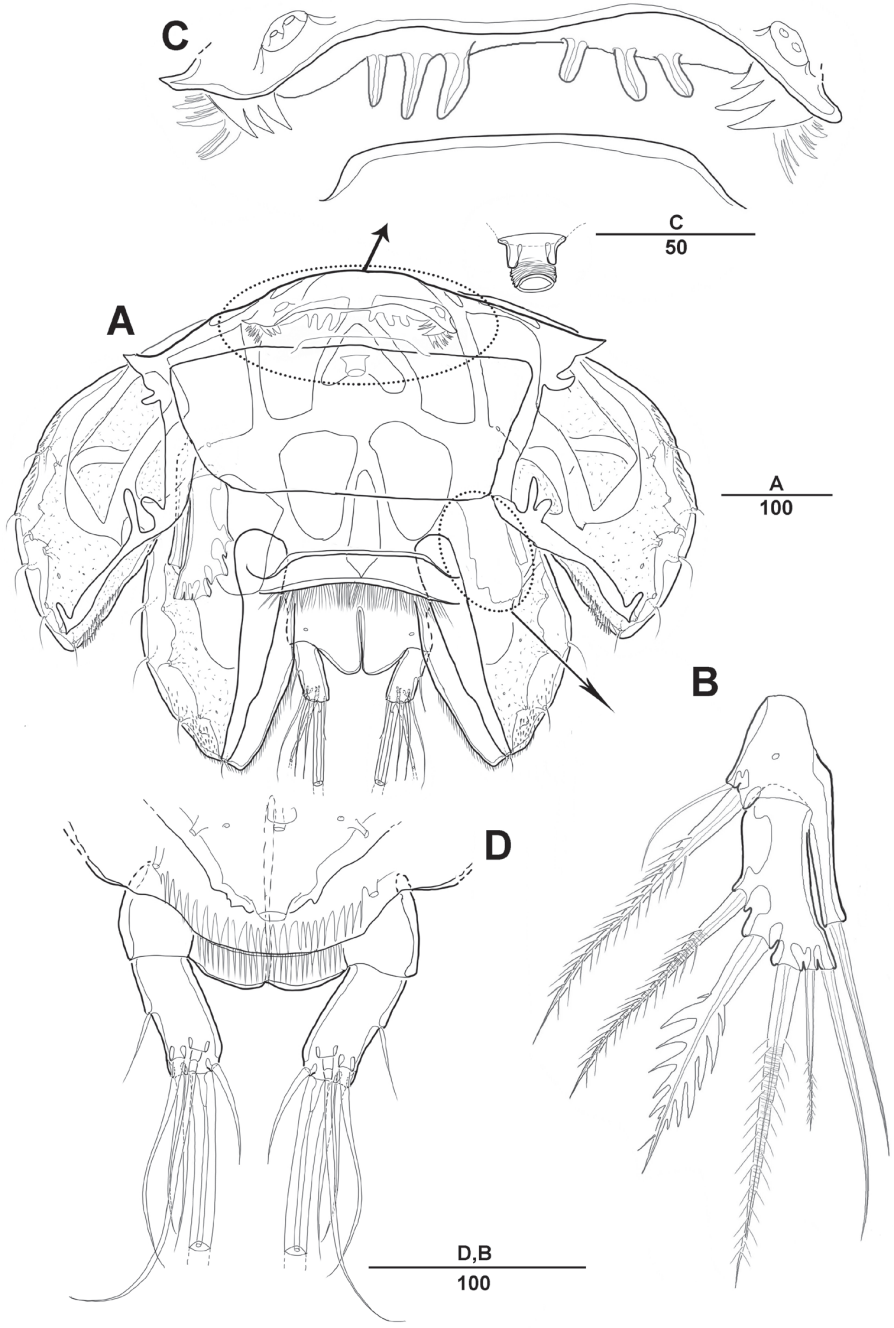
*Peltidium quinquesetosum* Song & Yun, 1999, female **A** urosomites, ventral **B** fifth thoracapod **C** genital field **D** caudal rami, dorsal. Scale bars indicate length in µm.

**Figure 22.**
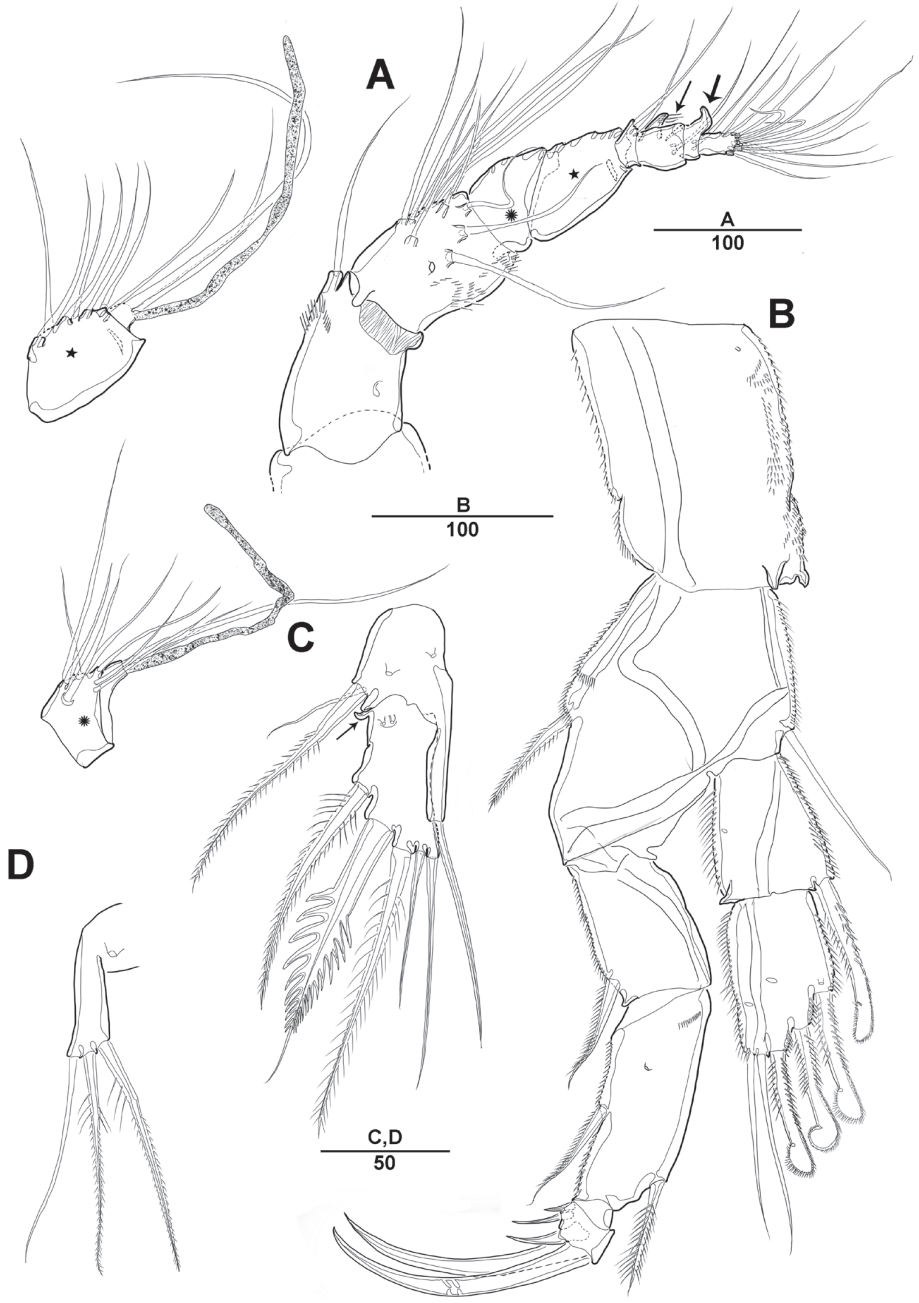
*Peltidium quinquesetosum* Song & Yun, 1999, male **A** antennule **B** first thoracapod **C** fifth thoracapod **D** sixth thoracapod. Scale bars indicate length in µm.

Figs 16 , 17 , 18 , 19 , 20 , 21 , 22

Peltidium quinquesetosum Song & Yun, 1999: 67–74, figs 1–3

####### Material examined.

Republic Of Korea (Table 1) ∙ 1 ♀ (MABIK CR00246774) was dissected on 10 slides ∙ 1 ♀ (MABIK CR00246775) was dissected on 6 slides ∙ 1 ♂ (MABIK CR00246787) was dissected on 10 slides ∙ 11 ♀♀ (MABIK CR00246776 to CR00246786) were preserved in 99% alcohol ∙ GenBank accession numbers: MT006218 to MT006229 (*COI*) and MT002903 to MT002905 (*18SrRNA*).

####### Note.

There was no remarkable difference between the original description and the specimens analyzed in the present study. However, additional details of sensilla on the surface, the structure of mouthparts and appendages, and the rows of spinules and setules were added in the figures.

## Discussion

### Relationships among *Diosaccus* spp.

The new species (*D. koreanus* sp. nov.) was placed in the genus *Diosaccus* on the basis of several characteristics (A2 exp with 4 setae, P2 exp-2 with 2 inner setae, P2 exp-1 without inner seta, and P4 enp 3-segmented) and was most closely related to *D. ezoensis* Itô, 1974, based on the setae formula of the swimming legs, mouthpart structures, and the shapes of P5 and P6. However, the new species was also clearly distinguishable from *D. ezoensis* based on the length of the second inner seta on the P5 exp (obviously longest in the female) and the presence of long setules along the outer margin of the P2 enp-3, as previously noted by Song et al. (1999). In addition, the present study found that *D. koreanus* sp. nov. could be further distinguished on the basis of caudal seta VII, which was located halfway from the rami base (vs. on anterior extremity in *D. ezoensis*), and P6 with 3 setae in the female (vs. 2 setae in *D. ezoensis*).

The genus *Diosaccus* currently contains 14 valid species (Bodin 1997; Wells 2007), one of which includes two subspecies and two of which are only placed in the genus provisionally. In addition, the latest dichotomous key (Lang 1965) for the genus used doubtful characters, like moderate length seta and the length of caudal rami, based on old manuscripts, and the tabular keys provided by Wells (2007) also include suspicious characters, such as the relative length between P1 enp-2 and enp-3, mainly owing to the lack of information about the species. Therefore, an updated key, which includes *D. koreanus* sp. nov., is presented below. Attempts were made to update the key on the basis of accurate characters. However, this was difficult because most of the original papers did not include full descriptions of the species. Because there is no apparent differentiation between *D. hamiltoni* and *D. tenuicornis* females, a single male character was added to the key. For species recorded before 1948 refer to the description of Lang (1948).

### Key to *Diosaccus* species, based mainly on female specimens

**Table:** 

1	A2 exp 3-segmented	**2**
–	A2 exp 2-segmented	**4**
–	A2 exp 1-segmented	**7**
2	P1 enp-2 without inner seta; basis of mxp robust	***D. rebus* (Sewell, 1940)**
–	Specimen without this combination of characters	**3**
3	Basis of mxp slender; P1 enp-3 longer than enp-2; P1 enp-2 with 1 inner seta	***D. valens* (Gurney, 1927)**
–	Base of mxp robust; P1 enp-3 as long as enp-2; P1 enp-2 without inner seta	***D. robustus* (Thompson & Scott, 1903)**
4	Seg-3 and seg-4 with sharp dorsal teeth; P5 exp with 7 setae; benp with 5 spines, nearly equal in length	***D. dentatus* (Thompson & Scott, 1903)**
–	Specimen without this combination of characters	**5**
5	P1 enp 2-segmented	***D. varicolor biarticulatus* (Monard, 1924)**
–	P2 enp 3-segmented	**6**
6	P5 exp with 6 setae	***D. varicolor varicolor* (Farran, 1913)**
–	P5 exp with 5 setae	***D. varicolor pentasetosus* (Noodt, 1955)**
7	P1 enp 2-segmented	***D. monardi* Sewell, 1940**
–	P1 enp 3-segmented	**8**
8	Benp with 6 setae/spines	**9**
–	Benp with 5 setae/spines	**11**
9	Caudal seta VII on proximally, P5 with 6 uniform (in length) setae, P6 with 2 setae	***D. ezoensis* Itô, 1974**
–	Specimen without this combination of characters	**10**
10	Second outer seta on P5 benp longest	***D. borborocoetus* Jakobi, 1954**
–	P5 benp with 6 spines	***D. koreanus* sp. nov.**
11	P5 benp with 5 spines	**12**
–	P5 benp with 5 spines/setae	**13**
12	Second outer seta on P5 benp longest; caudal seta II slender	***D. spinatus* Campbell, 1929**
–	First and second outer setae on P5 benp equal in length; caudal seta II strong	***D. truncates* Gurney, 1927**
13	P2 exp-3 with 3 outer spines; ♂ P5 benp with 2 setae, inner seta longer than outer seta	***D. hamiltoni* (Thompson & Scott, 1903)**
–	P2 exp-3 with 2 outer spines; ♂ P5 benp with 2 same length setae	***D. tenuicornis* (Claus, 1863)**

### Non-destructive DNA extraction and identification

The classification of harpacticoids has, until now, been primarily based on adult morphology, especially that of females. Significant differences between species, such as differences in number of segments or setae, are very important and recognizable characteristic that can be used to detect new species. However, some groups require researchers to classify species by features that are difficult describe, such as the width-to-length ratio of appendages, angle of segment inclination, and seta location. In addition, most of the recently discovered cryptic species are morphologically similar to known species. Although meiofauna are difficult to describe, owing to their small, fragile bodies, which make it difficult to obtain large amounts of genomic DNA from individual wild specimens (Sands et al. 2008), DNA sequencing can help with classification. The information about DNA sequences obtained from correctly classified species allows other researchers, for example, ecologists and researchers concerned with invasive species (Garrick et al. 2004) to quickly and easily classify species, even if they lack taxonomic knowledge. The use of DNA sequencing to identify and distinguish among cryptic species also allows taxonomists to identify more accurately taxonomically informative characteristics.

Previously identified harpacticoid species were described on the basis of morphological characteristics, not molecular ones. To classify benthic harpacticoids, observation is usually necessary under a high-power microscope. In this process, DNA in the specimen is destroyed by prolonged microscopic observation and the use of toxic media. Until now, it was difficult to get the DNA sequence and morphological information using same specimen. Therefore, there may be cases of incorrect registration of genetic information for other species. As in the present study and in Cornils (2015), the use of genetic information can reduce the error of species identification. However, specimen vouchers must be preserved for both the verification of genetic sequences and for morphological studies. The present study did not use genetic information for the phylogenetic analysis because the purpose of the study was to match accurately morphological features with the genetic information for each harpacticoid species. For an accurate phylogenetic study based on molecular and morphological data more species belonging to family Miraciidae are needed.

## Supplementary Material

XML Treatment for Diosaccus koreanus

XML Treatment for Parathalestris verrucosa

XML Treatment for Peltidium quinquesetosum
